# Specific symptom change associated with ecological momentary assessments of intrusive trauma memories

**DOI:** 10.1038/s44277-024-00019-4

**Published:** 2024-10-30

**Authors:** Yara Pollmann, Kevin J. Clancy, Quentin Devignes, Boyu Ren, Milissa L. Kaufman, Isabelle M. Rosso

**Affiliations:** 1 Division of Depression and Anxiety Disorders, McLean Hospital, Belmont, MA USA; 2 Department of Psychiatry, Harvard Medical School, Boston, MA USA; 3 Laboratory for Psychiatric Biostatistics, McLean Hospital, Belmont, MA USA

**Keywords:** Post-traumatic stress disorder, Outcomes research

## Abstract

As the global prevalence of exposure to traumatic events rises, there is a growing need for accessible and scalable treatments for trauma-related disorders like posttraumatic stress disorder (PTSD). Intrusive reexperiencing symptoms, such as trauma-related intrusive memories (TR-IMs), are central to PTSD and a target of gold-standard treatments that are effective but resource-intensive. This study examined whether completing a brief ecological momentary assessment (EMA) protocol assessing the occurrence and phenomenology of TR-IMs was associated with decreases in intrusion symptom severity. Trauma-exposed adults (N = 139) experiencing at least 2 TR-IMs per week related to a DSM-5 criterion A traumatic event completed a 2-week EMA protocol. During this period, they reported on 18 sensory-perceptual and affective qualities of their TR-IMs three times per day and on posttraumatic stress symptom severity at day’s end. Longitudinal symptom measurements were entered into linear mixed-effects models to test the effect of Time on symptom severity. Over the 2-week protocol, intrusion symptom severity decreased, while other symptom cluster scores did not change. Within the intrusion symptoms, this effect was specific to TR-IMs and emotional reactivity to trauma reminders, and was not moderated by survey completion rate, total PTSD symptom severity, ongoing treatment, or time since trauma. This study was quasi-experimental and lacked a control group, therefore no definitive conclusions about clinical utility can be made. Nonetheless, these findings provide preliminary proof-of-principle and warrant future clinical trials assessing the clinical efficacy of EMAs of intrusive trauma memories as a scalable treatment option targeting intrusive memory symptoms.

## Lay Summary

Exposure to trauma has risen globally, and unwanted memories are a common symptom that causes significant distress and impairment, yet often resists current treatments. In this study, we demonstrate that daily reporting on the unique qualities of trauma memories for two weeks was associated with decreases in trauma memory severity. These findings motivate future clinical trials to determine the efficacy and acceptability of daily smartphone-based monitoring of trauma memories as a novel, scalable intervention.

## Introduction

The global occurrence of traumatic events is rising steeply, with a growing prevalence of war, displacement, natural disasters, terrorism, violence, and other humanitarian crises. It is estimated that over 70% of the world’s population has been exposed to a traumatic event in their lifetime [1]. The impact of trauma at the individual, community, and societal levels is staggering. Data have showcased its association with increased risk of mental health disorders, low educational outcomes, and unemployment, exacting an annual economic toll of 3 billion in lost productivity in the United States alone [2, 3].

Existing guidelines for trauma-related psychiatric disorders, such as posttraumatic stress disorder (PTSD), list Cognitive Processing Therapy, Eye Movement Desensitization and Reprocessing, and Prolonged Exposure Therapy as the most empirically supported and recommended treatments [4], with these therapies consistently demonstrating safety and efficacy [5]. However, significant barriers to these modalities persist. These include high dropout rates and significant loss to follow-up undermining efficacy, shortage of trained therapists impeding accessibility, and sociocultural factors impacting acceptability [6–8]. Accordingly, there is a pressing need for innovative treatment approaches [9].

In addressing the need for novel therapies, there has been growing emphasis on targeting core, transdiagnostic symptoms that maintain distress and impairment, rather than solely focusing on categorical diagnoses [10]. While full remission of diagnostic syndromes like PTSD is often the goal, current therapies accomplish this in roughly one-third to one-half of patients who present for treatment [11, 12]. Even in those who achieve remission, many still meet criteria for at least one symptom cluster or report clinically significant residual symptoms, which may subsequently increase the risk for relapse [11, 13]. These shortcomings of therapeutic efficacy may be due, in part, to the marked heterogeneity of PTSD and its associated symptom structure, making it difficult to develop interventions that target the syndrome as a whole. This complexity is compounded when considering trauma exposure more comprehensively, as it often leads to adverse mental health outcomes beyond PTSD [14]. For instance, trauma-exposed individuals may exhibit only one or a few posttraumatic stress symptoms but experience significant distress and/or impairment nonetheless [15]. Moreover, they may experience a constellation of symptoms meeting diagnostic criteria for other psychiatric disorders [16]. Therefore, targeting mechanistically homogeneous symptom groupings or individual symptoms that are central to PTSD and other transdiagnostic post-traumatic psychiatric sequelae offers a promising avenue to enhance the efficacy of therapeutic interventions for trauma-exposed individuals [17–21].

Intrusive re-experiencing of the traumatic event is prevalent among trauma-exposed individuals, most commonly manifesting as trauma-related intrusive memories (TR-IMs). TR-IMs are involuntary, spontaneously surfacing and intruding on conscious thought processes [22]. They are characterized by their emotional intensity and sensory-rich detail, often emerging as images, smells, sounds, tastes, and bodily sensations [10, 17], as well as in the form of verbally accessible episodic memories [18]. Crucially, TR-IMs are key predictors of the development, severity, and maintenance of PTSD [23] and other adverse post-traumatic mental health outcomes [14], making them a critical therapeutic target for trauma survivors. On their own, they can cause clinically significant distress and impairment [10] and are often resistant to treatment [24]. Moreover, intrusion symptoms consistently represent a homogeneous symptom cluster in factor analytic studies [25] and the most central symptoms in network analyses [26–29], indicating shared mechanisms and a significant influence on other PTSD symptoms. Accordingly, intrusion symptoms as a group and TR-IMs individually are ideally suited for targeted therapies for trauma-exposed individuals [17–20].

Among the gold-standard treatments for PTSD, prolonged exposure (PE) therapy has the strongest focus on TR-IMs. PE accomplishes this through fear extinction, whereby patients repeatedly focus on recounting details of a traumatic experience in a safe environment until fear is reduced [30]. Despite their proven efficacy [31], PE and similar exposure therapies face significant challenges, including initial symptom exacerbation, high dropout rates, and dissemination barriers due to their dependence on highly-trained clinicians and environmental support [6, 7, 32]. Written exposure therapy (WET), a newer PTSD treatment grounded in exposure therapy principles, was developed to address these barriers by offering a brief, accessible protocol that involves repeated writing of trauma narratives [33]. Research shows that WET is non-inferior to PE [34], underscoring the clinical utility of brief, less resource-intensive protocols. However, despite these advances, barriers such as reliance on high-level clinician involvement and limited evidence for transdiagnostic utility persist, leaving many trauma-exposed individuals with TR-IMs without adequate intervention. This may be particularly relevant for individuals who do not meet diagnostic criteria for PTSD or whose presenting concerns are focused on repeated, intrusive trauma memories – individuals who may benefit from less intensive and briefer interventions [35].

Given these considerations, we conducted a secondary analysis of data from an observational study that utilized longitudinal assessments of TR-IMs to determine whether such repeated, detailed symptom monitoring is associated with changes in intrusion symptoms. Importantly, the present study was not designed as a prospective clinical trial with predetermined intervention or control groups. Instead, ecological momentary assessments (EMAs) were conducted daily to assess the phenomenological properties of TR-IMs, which entailed brief and repeated exposure to the sensory-perceptual and affective details of the trauma memory. Therefore, this project is viewed as a preliminary proof-of-principle study, without claims of direct clinical efficacy or utility. As a real-time data collection tool that periodically samples participants’ experiences [36], EMA has been used to track PTSD symptoms naturalistically, as was done in the current study. Prior research has examined the impact of EMA and related momentary assessments on symptom change, with some authors postulating that these repeated momentary assessments mirror some principles of exposure therapies [37–43]. However, no study has yet investigated the longitudinal effect of EMA on intrusive memory symptoms specifically, which may have critical transdiagnostic importance given the centrality of TR-IMs in post-traumatic psychiatric outcomes. Expanding upon this prior work, we hypothesized that completing this EMA protocol focused on TR-IMs would be associated with a specific reduction of intrusion symptom severity over time.

## Participants and methods

### Participants

Trauma-exposed adults (N = 220) were recruited via multiple sources as part of a larger investigation examining the neurobiological correlates of TR-IMs [44, 45]. The study was approved by the Mass General Brigham Human Research Committee, and all participants provided written informed consent. Inclusion criteria were: (a) 18-65 years of age, (b) exposure to at least one DSM-5 Criterion A trauma, (c) endorsement of at least two TR-IMs per week over the past month, (d) English proficiency, and (e) access to a “MetricWire” (MetricWire, Inc, Kitchener, Ontario) application-compatible smartphone. Exclusion criteria were: (a) current psychotic disorder or manic mood episode, (b) report of experiencing intrusions only as thoughts, as opposed to memories, (c) completion of less than 70% of the daily surveys during the EMA period, and (d) past month moderate-to-severe alcohol or substance use disorder.

Of the 220 enrolled participants, 35 did not complete all study procedures, 6 met diagnostic criteria for psychosis or a substance use disorder, and 1 did not endorse any TR-IMs during the survey period. Importantly, only 35 participants were excluded due to insufficient number of EMA surveys (15.9% non-compliance rate), and they did not differ from the final analyzed sample in total PTSD symptom severity or the severity of individual PTSD symptom clusters (p’s > .274), as assessed by the PCL-5 at baseline. Due to technical difficulties, 4 participants had survey periods extended beyond two weeks. Given our interest in the effect of Time for the analyses presented here, these participants were excluded, resulting in a final analyzed sample of n = 139. Table 1 shows participant demographic and clinical characteristics.

**Table 1:** Demographic and clinical characteristics.

Total Sample (N = 139)	
Age (years)	34.29 ± 11.20
Sex assigned at birth	
Female	102 (73%)
Male	37 (27%)
Gender	
Woman	89 (64%)
Man	35 (25%)
Non-binary	15 (11%)
Race	
White	100 (72%)
Multiracial^a^	19 (14%)
Black	9 (8%)
Asian	6 (5%)
Unknown or Not Reported	4 (1%)
Hispanic Ethnicity	
Non-Hispanic	129 (93%)
Hispanic	10 (7%)
Treatment (Psychotherapy or Medication)	87 (63%)
Baseline PCL-5 Item 1 (TR-IMs)	3.06 ± 0.84
Baseline PCL-5 Cluster B (Intrusion Symptoms)	12.73 ± 3.66
Baseline PCL-5 Total	47.99 ± 13.59
LEC-5 Total	13.24 ± 7.66
PTSD Diagnosis	106 (76%)
Number of completed TR-IM EMA surveys	35.91 ± 3.80
Number of completed PCL-5 EMA surveys	11.48 ± 2.12

*LEC-5* Life Events Checklist for DSM-5, *PCL-5* PTSD Checklist for DSM-5, *PTSD* Posttraumatic Stress Disorder, *TR-IM* Trauma-Related Intrusive Memory, *EMA* Ecological Momentary Assessment.

Mean ± standard deviation or N (%).

^a^Although “Middle Eastern / North African (MENA)” was not queried across all participants with a checkbox, 3 participants are counted in the multiracial category because they entered MENA as free text to indicate an additional race.

### Procedure

The study design consisted of two visits separated by a two-week EMA survey period. During Visit 1, participants provided written informed consent before initiating study procedures. They then completed self-report measures and received detailed instructions, along with a guided demonstration of the EMA smartphone application, provided by a non-clinician research assistant. Over the subsequent two-week EMA period, participants received five surveys daily. The first survey probed sleep quality and dreams/nightmares. The following three surveys assessed the occurrence and phenomenological properties of experienced TR-IMs (total number of surveys = 42). These surveys included adapted items from the Autobiographical Memory Questionnaire (AMQ) [38] to measure the vividness, visual features, sense of reliving, emotional intensity, fragmentation, and intrusiveness of TR-IMs, rated on a 0-4 Likert scale. The final survey assessed PTSD symptom severity over that past day (24 hours) using the Posttraumatic Stress Disorder Checklist (PCL-5; total number of surveys = 14). The survey schedule was personalized based on each participant’s sleep/wake times, and surveys were sent at semi-random times within 3-hour windows, beginning one hour before the participant’s typical wake time. Based on precedent in prior literature using EMA in individuals with serious mental illness, we established a cut-off threshold of 70% for survey completion to balance acceptability and feasibility with an adequate data return needed for our analyses [44, 46–48]. Therefore, only participants who completed at least 70% of all EMA surveys returned for Visit 2 to complete a clinical interview and neuroimaging.

### Measures

#### Life Events Checklist (LEC-5)

The LEC-5 [49] is a 17-item assessment of potentially traumatic events, reflecting a Criterion A trauma. The LEC-5 was used during Visit 1 to identify and orient participants to their Criterion A index trauma for EMA survey completion. In addition, total LEC-5 score was derived as an index of total lifetime trauma exposure for sample characterization (Table 1).

#### PTSD Checklist for DSM-5 (PCL-5)

The PCL-5 [50] was administered daily during the EMA period to assess PTSD symptoms. This 20-item self-report measure assesses the 20 DSM-5 symptoms of PTSD and is frequently used to monitor symptom change during and after treatment. While the PCL-5 typically assesses PTSD symptoms over the past month, we adapted it for our EMA protocol to query symptoms experienced over the past 24 hours, consistent with previous studies using daily adaptations [50, 51]. Each item is scored on a 0-4 Likert scale (0 = “Not at all” to 4 = “Extremely”). The PCL-5 provides a total PTSD symptom severity score (PCL-5 Total, Table 1) and PTSD symptom cluster scores: Intrusion (Cluster B), Avoidance (Cluster C), Negative alterations in mood and cognition (Cluster D), and Hyperarousal (Cluster E). Relevant to this study, the Intrusion symptom cluster includes 5 symptoms: (1) trauma-related intrusive memories (TR-IMs), (2) trauma-related nightmares or distressing dreams, (3) flashbacks, and (4) emotional and (5) physical reactions to trauma reminders.

#### Clinician-Administered PTSD Scale for DSM-5 (CAPS-5)

During Visit 2, the CAPS-5 [52] was administered by doctoral-level clinicians to determine PTSD diagnosis and clinician-rated symptom severity, including a score for total PTSD symptom severity (total CAPS-5 score).

#### Autobiographical Memory Questionnaire (AMQ)

The AMQ [53] is a 32-item questionnaire of autobiographical memory qualities, including categories of vividness, visual and auditory features, other sensory features, bodily sensations, language, emotions, perspective, reliving, fragmentation, and intrusiveness. An adaptation of AMQ items was utilized in the EMA surveys probing the phenomenological properties of TR-IMs, rated on a 0-4 Likert scale (0 = “Not at all” to 4 = “Definitely”).

### Statistical Analyses

To test the effect of Time on intrusive reexperiencing symptom severity, repeated measures of daily PCL-5 Intrusions (Cluster B) symptom scores over time were entered in a linear mixed effect model (LMM) using the lme4 package in R (Version 4.3.1) [54]. The model incorporated subject-specific random intercepts and slopes. The independent variable of Time reflects the timestamp of each completed PCL-5 survey in days relative to the beginning of the survey period. Age and sex were included as covariates, given their previously demonstrated roles in memory and PTSD symptoms [55–57].

To ascertain specificity of the effects to intrusion symptoms targeted in the EMA protocol, identical models were performed for the other PTSD symptom clusters (Avoidance, Mood/Cognition, Hyperarousal) and each individual intrusion symptom (intrusive memories, nightmares, flashbacks, emotional reactivity to reminders, physical reactivity to reminders). Multiple comparisons were adjusted using Bonferroni correction (across symptom clusters: 0.05/4 clusters = 0.0125; across intrusion symptoms: 0.05/5 symptoms = 0.010). Significant models surviving correction for multiple comparisons were re-run with the total number of completed memory surveys entered as an independent moderator of the effect of Time in separate models to examine a potential dose effect. Additional control analyses were performed with total CAPS-5 PTSD severity, ongoing treatment (psychotherapy or medication; binary 1/0), and time since trauma entered as independent moderators of the effect of Time in separate models to ascertain their potential influence on change in PTSD symptoms over time [58].

## Results

### Change in intrusion symptoms

**Fig. 1:**
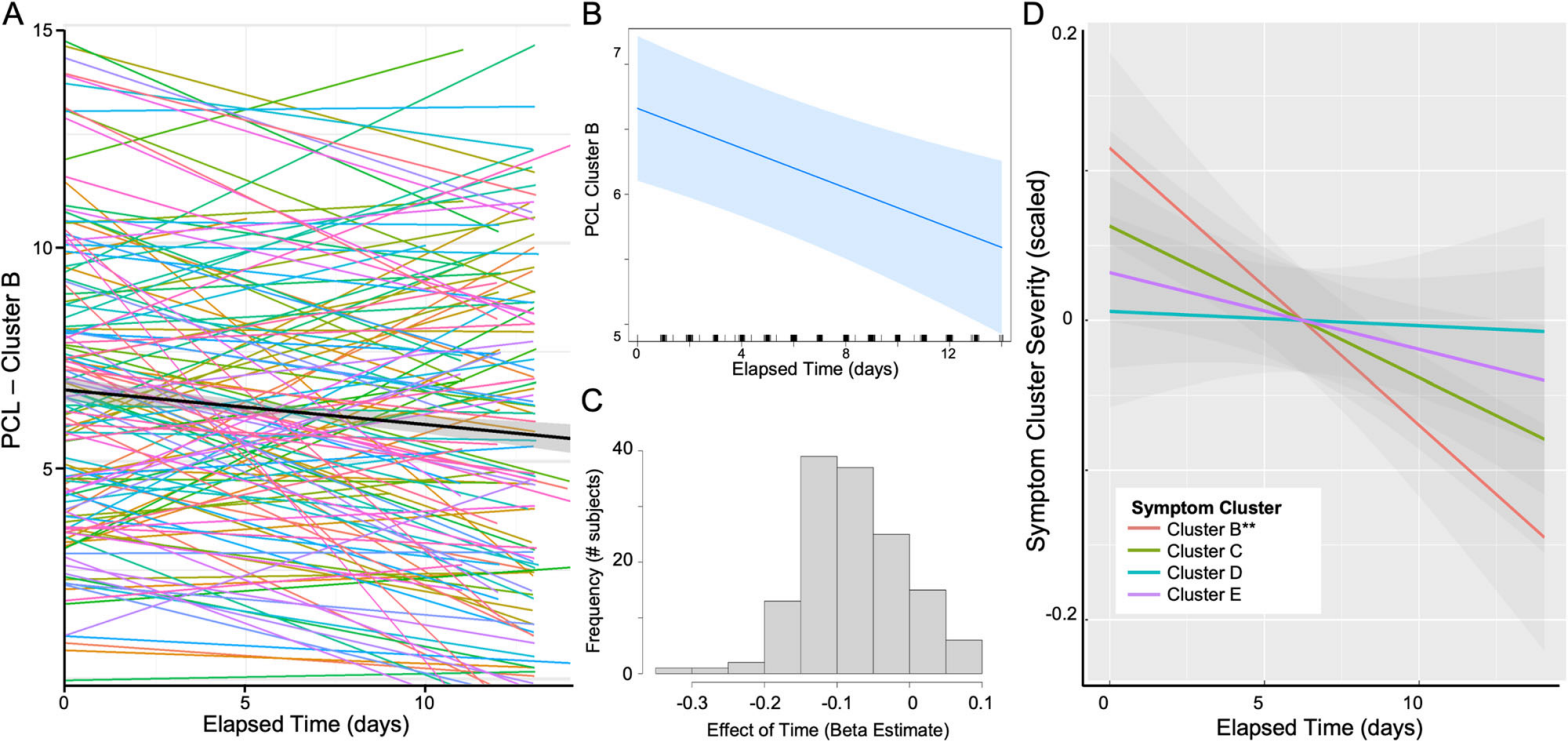
Effect of time on Cluster B—intrusive re-experiencing symptoms. **A** Individual lines represent subject-specific intercepts and slopes for the effect of time on PCL-5 Cluster B symptom severity score total with the group-level effect indicated by the black line and further depicted in (**B**). **C** Distribution of the effect of time across participants, demonstrating that most participants showed a decrease in Cluster B symptom severity scores over time. **D** Effect of time for each symptom cluster separately, demonstrating a significant effect on Cluster B score and no other symptom Cluster. Cluster scores were scaled in R (mean-centered and divided by standard deviation) to allow for uniform comparison across symptom clusters. **p < 0.001.

The linear mixed effects model predicting PCL-5 Cluster B severity revealed a significant effect of Time on daily intrusion symptoms (beta = −0.08, t = −3.48, p < 0.001), with participants demonstrating a decrease in intrusion symptom severity over the EMA period (Fig. 1A–C). This effect was independent of the total number of completed memory surveys (p = 0.448). Identical linear mixed models performed with other PCL-5 symptom clusters revealed no effect of Time for Cluster C (avoidance; t = −1.45, p = 0.150), Cluster D (mood/cognition; t = −0.35, p = 0.730), or Cluster E symptoms (hyperarousal; t = −0.95, p = 0.344; Fig. 1D).

### Specificity of symptom change to intrusive memories

**Fig. 2:**
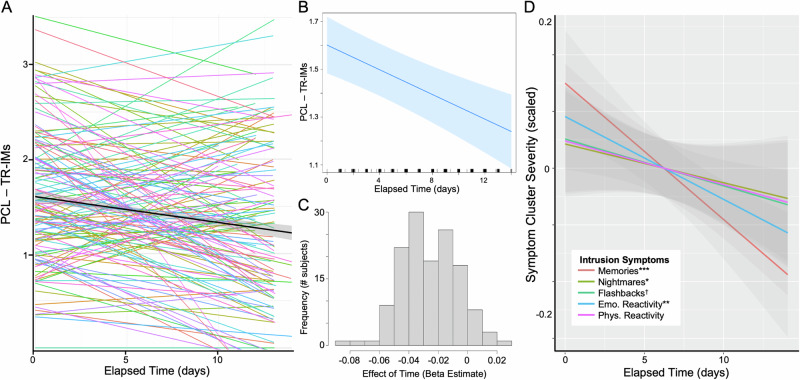
Effect of Time on Trauma-Related Intrusive Memories (TR-IMs). **A** Individual lines represent subject-specific intercepts and slopes for the effect of time onPCL-5 TR-IMs severity score, with the group-level effect indicated by the black line and further depicted in (**B**). **C** Distribution of the effect of time across participants, demonstrating that most participants showed a decrease in TR-IMs over time. **D** Effect of time for each Cluster B symptom separately, demonstrating a significant effect on TR-IMs and no other Cluster B symptom. Scores were scaled to allow for comparison across symptoms. †p < .10, *p < .05, **p < .005, ***p < .001.

Specificity to individual TR-IM symptoms was examined through additional linear mixed models. A significant effect of Time was seen on TR-IMs (beta = −0.03, t = −4.59, p < 0.0001; Fig. 2A–C) and emotional reactivity to trauma reminders (beta = −0.02, t = −3.20, p = 0.002). There was a small effect of Time on nightmares (beta = −0.01, t = −2.03, p = 0.045), which did not survive correction for multiple comparisons, and a marginal effect on flashbacks (beta = −0.009, t = −1.82, p = 0.070). There was no effect on physical reactivity to trauma reminders (t = −1.62, p = 0.108). The effects of Time on TR-IMs and emotional reactivity to reminders were independent of the total number of memory surveys completed (p = 0.616).

### Control analyses

Our results were robust to the influence of clinical characteristics that may impact PTSD symptoms over time. There was no effect of total CAPS-5 PTSD severity on the observed change in TR-IM severity (p = 0.819). Additionally, there was no effect of ongoing treatment (p = 0.655) or time since trauma (p = 0.616) on the observed change in TR-IM severity.

## Discussion

This study examined an EMA protocol assessing TR-IM occurrence and features in relation to changes in posttraumatic stress symptom severity, providing preliminary evidence that completing this EMA protocol is associated with reductions in intrusive reexperiencing symptom severity. We observed no change in the severity of avoidance symptoms, negative alterations in cognition and mood, or alterations in arousal and reactivity, highlighting a specificity of this effect to intrusion symptoms. Additional specificity analyses revealed that the decrease in intrusion symptom severity was specific to a change in TR-IMs – the focus of the EMA protocol – as well as emotional reactivity to trauma reminders. These findings provide preliminary evidence supporting the tenet that an EMA protocol assessing the phenomenology of TR-IMs may reduce intrusion symptoms and related emotional distress.

Critically, the present study was not designed as a clinical trial to provide evidence for the clinical utility or efficacy of the EMA protocol. The data originate from an individual difference (within-subjects) study conducted for non-treatment purposes, without control groups or measures of mechanisms of change. Therefore, no definitive conclusions can be drawn about the efficacy of the EMA protocol, as the observed symptom changes could reflect chance or regression to the mean over time. Acknowledging this critical limitation, the specificity of the observed reductions to the intrusive trauma memory symptoms targeted by the EMA protocol, alongside a more stable trajectory of other symptoms, offers preliminary evidence that this EMA protocol may achieve meaningful symptom change. Indeed, the magnitude of reduction observed for the TR-IM symptom fell within the proposed range of “minimally important change” [59], although applying this metric to a single symptom should be interpreted with caution. We believe these findings provide important yet preliminary proof of principle for the use of EMAs to target TR-IMs in an accessible and transdiagnostic way. Below we discuss possible explanations for the observed specific symptom change and propose candidate mechanisms of change that are in line with prior work using EMA or self-monitoring protocols.

Our findings align with and build upon previous research demonstrating the therapeutic efficacy of self-monitoring for PTSD symptoms. EMA protocols are typically used as a data collection or monitoring tool, with less focus on their potential effect on symptom severity. Nonetheless, research has indicated that daily EMA surveys simply monitoring PTSD symptoms in a naturalistic setting can lead to significant reductions in total PTSD symptom severity [38], setting the precedent that monitoring symptoms may result in their improvement. Consistent with this notion, a series of prior studies have demonstrated improvements in general PTSD symptoms following daily self-monitoring of intrusion symptoms in PTSD patients. However, these studies were limited to 4 or 5 questions about the number, content, and intensity of the intrusion symptoms and included weekly outpatient visits with study staff to review the monitoring diaries [42, 43]. In our study, the EMA protocol went beyond mere symptom monitoring by prompting participants to recall in detail 18 different sensory-perceptual and emotional properties of their TR-IMs, potentially fostering greater engagement with and processing of these memories [60, 61]. Additionally, we found that the effect of the EMA protocol was specific to the TR-IM symptom, unlike the prior studies that demonstrated broader symptom improvement across other PTSD symptom clusters. This highlights the targeted nature of the EMA protocol and provides some evidence, albeit inconclusive, that the observed symptom changes were not due to chance.

When considering the potential active components of self-monitoring or EMA protocols that lead to symptom change, prior work has proposed that the frequent and regular reporting of symptoms may mirror elements of exposure therapies [38]. However, it is critical to acknowledge the marked differences between such self-monitoring protocols and true exposure therapies. Exposure-based interventions, such as PE, require detailed and sustained activation of the explicit content of the traumatic experience over extended periods of time to achieve habituation. Additionally, exposure therapies include clinician-guided debriefings and processing of each exposure. When combined, these elements activate the “fear structure” [62], the neurocognitive state that represents a response to learned fear, and facilitate emotional processing of trauma memories that may lead to lasting habituation. This positions emotional processing of and habituation to activated fear structures as critical active components of exposure therapies [62, 63].

Our protocol did not explicitly prompt the recounting of the traumatic event, nor did it involve direct or guided processing of the trauma memories. However, the repeated reporting of detailed sensory-perceptual properties of TR-IMs in our EMA protocol may have been sufficient to reactivate the memory – an interpretation founded in prior work utilizing the same prompts about memory details [60, 61]. Research with healthy participants has demonstrated that completing the AMQ in reference to a negative memory can reduce emotional reactivity and distress, which the authors conceptualized as a consequence of extinction due to exposure to details of the trauma memory [60, 61]. For example, participants are asked questions such as: “I could describe where the people and things were located”; “I could identify the people and things in the memory”; “I experienced a scene in which elements were located relative to each other”; and “I could identify or name the setting where the memory occurred”. The authors suggest that responding to these questions requires participants to “reinstate” or mentally reconstruct the memory in detail which, in their interpretation, “activates” the reliving of the memory sufficiently to elicit a therapeutic response [60, 61]. Dewey et al. [38] suggest that this process could activate the ‘fear structure’ and facilitate emotional processing of the trauma memories. Our findings of reduced emotional reactivity support this notion, potentially indicating some degree of indirect emotional processing. Moreover, the repeated imaginal reliving of memories multiple times per day may promote habituation, leading to reduced reactivity to cues or reminders. Therefore, consistent with these prior studies, emotional processing and habituation are potential candidate mechanisms for the observed reductions in intrusion symptoms in the current protocol. However, it is unknown whether or to what extent our EMA protocol reactivated the trauma memory to engage the fear structure and elicit subsequent emotional processing or habituation. Future RCTs with validated measures of mechanisms of change are needed to determine whether repeated EMAs of TR-IMs engage similar active components as exposure-based therapies.

There have been significant efforts to develop therapeutic interventions that incorporate the active components of exposure therapies while addressing the limitations and barriers faced by many patients and clinicians. For example, written exposure therapy (WET) was designed as a less intensive, shorter- duration intervention that does not rely on extensive clinician involvement [34]. Additional approaches, such as internet-based cognitive behavioral therapy [64, 65], have been developed to address personnel demands of evidence-based interventions, with robust evidence for effectiveness of adaptations that minimize clinician involvement. Recent studies have demonstrated non-inferiority of an internet-based adaptation of WET that incorporates peer specialists instead of clinicians [34]. This is a critical barrier to address given unequal access to trained mental health professionals [66] and clinician hesitancy to offer exposure-based therapies [67]. Nonetheless, there is still a critical role for trained personnel or clinicians to play in true exposure therapies, such as facilitating the appropriate engagement in exposures and guiding post-exposure processing. Acknowledging the present EMA protocol is not a true exposure, it was highly accessible and feasible for participants to complete in a self-directed manner with no involvement of trained study staff or clinicians. Therefore, if future studies confirm engagement of exposure mechanisms by the EMA protocol, it could offer an avenue for individuals to engage with some active components of exposure therapy in a self-guided manner.

While the specificity of symptom reduction to TR-IMs offers potential evidence that the observed changes are not due to chance, it also presents a shortcoming compared to gold-standard therapies that address all symptom clusters. As discussed, most exposure-based interventions involve post-exposure processing to consolidate and generalize what is learned from the exposure. This additional processing may drive improvements in generalized hyperarousal, alterations in mood and cognition, or avoidance of trauma reminders. Furthermore, most evidence-based protocols span 8 to 15 weeks [68], though some massed or condensed versions have been implemented [69]. Given the temporal dynamics of trauma symptoms and the strong predictiveness of intrusion symptoms in driving other symptom clusters [29], it is reasonable to hypothesize that extending this EMA protocol beyond two weeks in future studies could lead to symptom alleviation in other clusters.

Overall, future RCTs are needed to evaluate the clinical efficacy of our EMA protocol for TR-IMs and the mechanisms driving intrusion symptom reduction. These trials should collect detailed information on recent or ongoing evidenced-based therapies, particularly exposure-based interventions, and should identify predictors of symptom change. The inclusion of detailed assessments of the active elements of exposure therapy, such as emotional processing and habituation, would determine whether the EMA protocol engages similar established mechanisms of symptom change. Although our results were robust to controlling for ongoing psychotherapy, we did not collect information about the duration or type of therapy. It is possible that synergistic effects occur in individuals who have completed or are currently completing exposure therapy if the EMA protocol is, in fact, engaging similar mechanisms of change. They are characterized by their emotional intensity and sensory-rich detail, often emerging as images, smells, sounds, tastes, and bodily our results were robust to controlling for clinician-rated PTSD severity, in-depth examination of baseline symptom presentations may help identify who could benefit most from this EMA protocol. For example, this protocol may offer a brief, scalable intervention for less severely symptomatic individuals on waitlists, those who do not meet full diagnostic criteria for PTSD, or individuals who might benefit from less intensive interventions [35, 70]. Relatedly, although we did not observe a dose-response relationship, the nature of our inclusion criteria (a minimum of 70% survey completion) may have hindered appropriate analyses of dose effects. Therefore, future RCTs should examine when individuals achieve clinically significant symptom change and how long these changes persist. This consideration is particularly important given the potential burden of daily interventions for some individuals. The high data return rate and low non-compliance rate observed in our study suggest that the current protocol was both feasible and acceptable, and that the observed change in TR-IM severity may meet criteria for minimally important change [59]. Nonetheless, future RCTs should include direct measures of acceptability and feasibility to determine at what point the burden of repeated EMA surveys might outweigh the potential benefits of achieving clinically significant symptom change.

Following validation of the preliminary clinical efficacy of an EMA protocol for TR-IMs through an RCT, there are several promising future directions. One possibility is to develop the EMA protocol into an ecological momentary intervention (EMI) framework by adapting or including additional prompts based on reported TR-IM qualities, which may enhance clinical efficacy by increasing engagement and processing of TR-IMs. Additionally, there is potential for clinical utility across psychiatric disorders, given the role of intrusive memories in multiple different conditions [71–73]. There is also substantial need to address subthreshold posttraumatic stress [15], as many evidence-based treatments are predicated on individuals meeting full diagnostic criteria for PTSD [4]. By targeting TR-IMs, we could reduce the risk of additional symptoms developing and progressing into full PTSD or other trauma-related disorders, given the centrality of intrusion symptoms [74, 75]. Additionally, because this protocol was conducted entirely electronically via smartphone, it offers potential for widespread therapeutic use. This is bolstered by the adaptability, accessibility, cost-effectiveness, and cultural sensitivity that such technology-based mental health care offers [76]. While technology-based barriers exist, rapidly increasing technology use and access in both developed and developing countries provides a promising avenue for expanding these interventions [77–79]. Studies have shown the acceptability of using EMA and EMI approaches in lower-income groups in the US and across lower- and middle-income countries [80, 81]. Incorporating community-based participatory methods would help tailor these technology-based approaches to meet the unique needs of diverse populations.

In conclusion, this study provides preliminary proof-of-principle evidence for changes in trauma memory severity following completion of an EMA protocol assessing the occurrence and properties of trauma memories. Future RCTs are needed to conclude on the clinical utility, efficacy, and acceptability of this EMA protocol and to elucidate the active components driving symptom change. If proven effective, extensions of this EMA protocol could offer a scalable, accessible, and culturally sensitive approach to addressing intrusive memories, a central symptom of trauma-related disorders. Given that traumatic exposure is ballooning globally, such scalable protocols may play a crucial role in reducing the burden of trauma-related psychiatric disorders worldwide, advancing the movement towards technology-based care and improving access to effective treatment for all.

### Citation diversity statement

The authors have attested that they made efforts to be mindful of diversity in selecting the citations used in this article.

## Data Availability

Data are available upon reasonable request to the corresponding author (IMR) and following the execution of a data use agreement.
